# Genotyping-by-sequencing data of 272 crested wheatgrass (*Agropyron cristatum*) genotypes

**DOI:** 10.1016/j.dib.2017.09.030

**Published:** 2017-09-22

**Authors:** Pingchuan Li, Bill Biligetu, Bruce E. Coulman, Michael Schellenberg, Yong-Bi Fu

**Affiliations:** a Department of Plant Sciences, University of Saskatchewan, 51 Campus Drive, Saskatoon, Saskatchewan, Canada S7N 5A8; b Semiarid Prairie Agricultural Research Centre, Agriculture and Agri-Food Canada, Swift Current, Saskatchewan, Canada S9H 3X2; c Plant Gene Resources of Canada, Saskatoon Research and Development Centre, Agriculture and Agri-Food Canada, Saskatoon, Saskatchewan, Canada S7N 0X2

**Keywords:** Crested wheatgrass, Genotyping-by-sequencing, Diploid, Tetraploid, Raw sequence data

## Abstract

Crested wheatgrass [*Agropyron cristatum* L. (Gaertn.)] is an important cool-season forage grass widely used for early spring grazing. However, the genomic resources for this non-model plant are still lacking. Our goal was to generate the first set of next generation sequencing data using the genotyping-by-sequencing technique. A total of 272 crested wheatgrass plants representing seven breeding lines, five cultivars and five geographically diverse accessions were sequenced with an Illumina MiSeq instrument. These sequence datasets were processed using different bioinformatics tools to generate contigs for diploid and tetraploid plants and SNPs for diploid plants. Together, these genomic resources form a fundamental basis for genomic studies of crested wheatgrass and other wheatgrass species. The raw reads were deposited into Sequence Read Archive (SRA) database under NCBI accession SRP115373 (https://www.ncbi.nlm.nih.gov/sra?term=SRP115373) and the supplementary datasets are accessible in Figshare (10.6084/m9.figshare.5345092).

**Specifications Table**

**Table:** 

Subject area	*Agricultural and biological sciences*
More specific subject area	*Plant genomics*
Organism	*Crested wheatgrass* [*Agropyron cristatum* L. (Gaertn.)]
Type of data	*Genotyping-by-sequencing data*
How data was acquired	*Illumina MiSeq on GBS libraries*
Data format	*Raw reads in FASTQ format and meta data in plain text*
Experimental factors	*Crested wheatgrass plants grown in greenhouse*
Experimental features	*gd-GBS protocol*
Data source location	*Agriculture and Agri-Food Canada and University of Saskatchewan*
Data accessibility	*Raw sequence data are available from NCBI SRA with accession SRP115373 and meta data are accessible from Figshare*

**Value of the data**

• The first large set of next generation sequencing datasets obtained from genomic DNAs for 272 diploid and tetraploid crested wheatgrass plants. These plants represent seven breeding lines, five cultivars and five geographically diverse accessions.
• These datasets can be utilized to enhance genetic and genomic studies, genetic diversity assessments, and marker-assistedbreeding of crested wheatgrass.
• The SNP datasets can be directly explored for the development of useful molecular markers to investigate genetic variability of crested wheatgrass and to facilitate the molecular breeding of this plant.

## 1 Data

Our sequencing efforts in crested wheatgrass generated two new sets of genomic data. The first set consists of 608 FASTQ files generated for 272 diploid and tetraploid crested wheatgrass plants representing seven breeding lines, five cultivars and five geographically diverse accessions (see Table 1). These sequences were obtained from genomic DNA through genotyping-by-sequencing (GBS) technique using an Illumina MiSeq instrument for seven runs with paired-ends of 250 bp in length. Plants from two accessions were sequenced twice as a control for quality assessment. Raw reads for all 17 accessions were deposited into NCBI's SRA database with accession number SRP115373 (https://www.ncbi.nlm.nih.gov/sra?term=SRP115373). The second set contains several meta data files generated from bioinformatics analysis of the sequence reads, including contigs for diploid and tetraploid plants and SNPs for diploid plants. These supplementary datasets have been deposited in Figshare (10.6084/m9.figshare.5345092).

**Table 1:** List of 17 crested wheatgrass accessions used in the study.

**Accession**	**CN number**^a^	**Alternative identification**^a^	**Origin**	**Ploidy**
AC Parkland	–	FOR483	Canada	2x
Fairway	CN32968	FOR533	Canada	2x
PGR 16452	CN43215	–	Kazakhstan	2x
PGR 16454	CN43217	–	Iran	2x
S9542	–	S9542	Canada	2x
AC Goliath	CN108673	–	Canada	4x
AC Newkirk	–	FOR552	Canada	4x
Karabalykskij 202	CN31068	–	Kazakhstan	4x
Kirk	CN108662	–	Canada	4x
PGR 16830	CN43478	–	Kazakhstan	4x
S8959E	–	FOR917	Canada	4x
S9491	–	S9491	Canada	4x
S9514	–	S9514	Canada	4x
S9516	–	S9516	Canada	4x
S9544	–	S9544	Canada	4x
S9556	–	S9556	Canada	4x
Vysokij 9	CN30995	–	Siberia	4x

a CN number is the accession identification in Plant Gene Resources of Canada, Agriculture and Agri-Food Canada (AAFC), while the alternative accession labels including FOR or S are from the joint forage breeding program of the University of Saskatchewan and AAFC.

## 2 Experimental design, materials and methods

### 2.1 Plant materials and DNA extraction

This study selected 17 crested wheatgrass accessions consisted of seven breeding lines, five cultivars and five geographically diverse accessions (Table 1). Five accessions are diploid and 12 accessions are tetraploid. These accessions were acquired from USDA plant germplasm system, Plant Gene Resources of Canada, and the joint forage breeding forage program of the University of Saskatchewan and Agriculture and Agri-Food Canada. Seeds were randomly selected from each accession and grown for six weeks in a greenhouse at the Saskatoon Research and Development Centre, Agriculture and Agri-Food Canada, under a 12 h photoperiod at 25 °C during the day time. Young leaf tissues were collected from 16 randomly selected plants of each of the 17 accessions, and stored at −80 °C prior to DNA extraction. For each of the 272 samples, DNA was extracted from 0.1 g ground tissue by following the protocols of NucleoSpin® Plant II Kit (Macherey-Nagel, Bethlehem, PA, USA) and eluted in a 1.5-ml Eppendorf tube with Elution Buffer. The DNA quality was measured with NanoDrop 8000 (Thermo Scientific) by comparing the 260 and 280-nm absorption. DNA samples were further quantified through the Quant-iTTM PicoGreen® dsDNA assay kit (Invitrogen) and subsequently diluted to 60 ng/μl with 1×TE buffer prior to sequencing analysis.

### 2.2 GBS library preparation and sequencing

The complexity reduced and multiplexed GBS libraries were prepared following the published gd-GBS protocol [1]. In brief, each library preparation started with 200 ng of purified genomic DNA by restriction enzyme digestion of *PstI* + *MspI*. Ligations between specifically customized 5′/3′ adapters and inserts by T4 ligase were carried out using standard product protocol. Ligated fragments were purified by AMPure XP kit and subsequently amplified and indexed with Illumina TruSeq HT multiplexing primers. Six library pools were made and each consisted of 48 indexed samples (3 accessions×16 individual plants). One extra library pool was included using 32 samples from two randomly selected accessions as a control. Prior to pooling of samples into a library, amplicon fragments from TruSeq HT kits were pre-selected by Pippin instrument for an insert size ranging between 250 and 450 bp, but the actual fragment size varied between 400 and 600 bp. Each pooled library was diluted to 6pM and denatured with 5% of sequencing-ready Illumina PhiX Library Control that serves for calibration for sequencing confidence. Sequencing was performed at the Saskatoon Research and Development Centre using an Illumina MiSeq instrument with paired-ends of 250 bp in length. Seven MiSeq runs generated 608 FASTQ sequence files for 272 plants from 17 accessions. Note that 32 plants from two accessions were sequenced twice as control for quality assessment.

### 2.3 Contig assembly and sequence similarity analysis

Contig sequence was assembled by using protein associated SNP prediction and genotyping pipeline paSNPg [2], which was specifically developed for non-model species and requires two inputs including the raw MiSeq sequence reads and relevant plant Ensembl PEP package [3]. Pep_database.tar.bz2 tarball was prepared by following the protocols of paSNPg to merge 44 plants species’ PEP data and was placed together with paired-end sequence reads (or FASTQ files) as a combined input for paSNPg. The default settings were adopted for *k*-mer size (100 bp) and minimal percentage of sample size (MPSS, 80%). Contigs were generated mainly through the Minia routine [4] implemented in the paSNPg pipeline. The analysis generated 6674 contigs for diploid crested wheatgrass plants with the default setting of parameters: 75% of identical match and 99% of alignment length. Among those contigs, 768 (11.5%) were associated with exons of coding genes. A total of 7792 contigs were assembled for tetraploid crested wheatgrass plants, while 809 (10.3%) assembled contigs were associated with exons of coding genes.

Efforts were also made to assemble contigs for separate diploid or tetraploid accessions, following the same parameter setting as for the combined analysis of diploid or tetraploid plants. The outcomes are summarized in Table 2. A sequence similarity analysis of these contigs was also made between diploid and tetraploid plants using Blastn search among diploid- and tetraploid-based contigs under the cut-off of 1e-100 for E-value [5]. The parsed results revealed 4477 (67%) diploid-based contigs sequences matched with 4461 (57%) tetraploid-based contigs.

**Table 2:** The MiSeq sequence data profile and empirical genomic coverage (EgC) for 17 crested wheatgrass accessions.

**Accession**^a^	**Raw reads**	**Trimmed reads**	**With 5′ Hinfl residues**^b^	**Contig×length (bp)**^c^	**Total contig length (bp)**	**EgC (%)**^d^	**Biosample accession**
AC Parkland	6,823,899	5,968,413	5,948,980	12,749×239	3,057,232	0.044	SAMN07502767 – SAMN07502782
Fairway	8,838,311	7,023,400	5,312,458	18,204×240	4,376,232	0.063	SAMN07502815 – SAMN07502846
PGR16452	8,415,793	6,919,568	5,252,776	34,236×241	8,283,160	0.120	SAMN07502847 – SAMN07502878
PGR16454	7,750,962	6,400,207	6,070,265	27,981×241	6,750,278	0.098	SAMN07502879 – SAMN07502894
S9542	7,905,221	6,248,364	6,048,368	18,528×235	4,371,062	0.063	SAMN07502991 – SAMN07503006
AC Goliath	7,084,059	5,981,098	5,961,130	12,227×241	2,947,930	0.022	SAMN07502735 – SAMN07502750
AC Newkirk	6,832,714	5,621,605	5,605,880	9471×239	2,265,760	0.017	SAMN07502751 – SAMN07502766
Karabalykskij 202	6,826,807	5,412,874	5,134,977	8754×238	2,089,266	0.015	SAMN07502799 – SAMN07502814
Kirk	6,103,771	5,172,052	5,153,987	8580×241	2,069,305	0.015	SAMN07502911 – SAMN07502926
PGR16830	7,208,410	5,978,683	5,668,841	10,722×238	2,560,384	0.019	SAMN07502895 – SAMN07502910
S8959E	6,312,324	5,373,354	5,353,217	9546×241	2,300,750	0.017	SAMN07502927 – SAMN07502942
S9491	8,412,288	6,951,698	6,910,099	14,807×240	3,563,450	0.026	SAMN07502943 – SAMN07502958
S9514	8,194,719	6,883,223	6,836,397	9328×240	2,238,810	0.017	SAMN07502959 – SAMN07502974
S9516	8,010,454	6,687,373	6,635,121	11,730×240	2,815,457	0.021	SAMN07502975 – SAMN07502990
S9544	8,496,678	7,132,507	6,896,082	16,800×236	3,968,754	0.029	SAMN07503007 – SAMN07503022
S9556	7,440,684	6,235,134	6,026,824	17,085×237	4,050,705	0.030	SAMN07503023 – SAMN07503038
Vysokij 9	6,874,703	5,872,351	5,855,738	12,528×239	2,999,840	0.022	SAMN07502783 – SAMN07502798

a Only the forward (R1) sequence reads from all individual plants of each accession were used for contig assembly.

b The number of sticky ends of *PstI* 'TGCA' found in 5′ end.

c The length representing the average contig length in base pairs.

d Based on the average genome size of crested wheatgrass estimated through flow cytometry with those of *Triticum durum* and *Triticum aestivum*.

Additional analysis was made to assess the gd-GBS empirical genome coverage (EgC) for each accession. The genome sizes of crested wheatgrass plants were estimated through flow cytometry based on the genome sizes of *Triticum durum* and *Triticum aestivum*. The average genome size of 6898 Mbp and 13,527 Mbp were obtained for diploid and tetraploid plants, respectively. The EgC values ranged from 0.044% to 0.120% with a mean of 0.078% for diploid plants, and ranged from 0.015% to 0.030% with an average of 0.021% for tetraploid plants (Table 2).

### 2.4 Protein-associated SNP identification

Efforts were made to do a SNP call only for diploid plants using the paSNPg pipeline, as it was developed specifically for diploid non-model species. Currently, there is no effective pipeline available for SNP calls from genomic sequences of tetraploid plants. A total number of 11,854 nuclear SNPs were successfully discovered from diploid plants, of which 1738 (14.7%) SNPs were associated with exons of coding genes. However, the number of total nuclear SNP and exon-associated SNPs without missing values across diploid plants were smaller with 1158 and 308, respectively.
