# Supplementary material for: Genotyping-by-sequencing data of 272 crested wheatgrass (Agropyron cristatum) genotypes
Source: Data Brief. 2017 Sep 22;15:401–6. doi: 10.1016/j.dib.2017.09.030 (PMC5712052; doi:10.1016/j.dib.2017.09.030)
Supplement: Supplementary file 1 — Supplementary material [file mmc1.pdf]

AUTHOR DECLARATION OF CONFLICT OF INTEREST

We wish to confirm that there are no known conflicts of interest associated with this publication and there has been no significant financial support for this work that could have influenced its outcome.

We confirm that the manuscript has been read and approved by all named authors and that there are no other persons who satisfied the criteria for authorship but are not listed. We further confirm that the order of authors listed in the manuscript has been approved by all of us.

We understand that the Corresponding Author is the sole contact for the Editorial process (including Editorial Manager and direct communications with the office). He/she is responsible for communicating with the other authors about progress, submissions of revisions and final approval of proofs.

Signed by the corresponding author on behalf of all authors

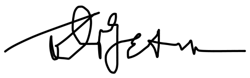

Sep 6 2017

AUTHORS:

Pingchuan Li
Bill Biligetu (Corresponding author)
Bruce E. Coulman
Michael Schellenberg
Yong-Bi Fu
